# Visceral leishmaniasis in an adult case in Northeast of Iran: A case report and literature review

**DOI:** 10.1002/ccr3.3303

**Published:** 2020-10-01

**Authors:** Mohammad Reza Taghavi, Samaneh Mollazadeh, Mohsen Seddigh‐Shamsi, Amir Azimian, Mahnoush Mianji, Mina Sadat Mohajerzadeh Heydari, Zahra Zandi, Azar Shokri

**Affiliations:** ^1^ Faculty of Medicine North Khorasan University of Medical Sciences Bojnurd Iran; ^2^ Natural Products and Medicinal Plants Research Center North Khorasan University of Medical Sciences Bojnurd Iran; ^3^ Department of Hematology Oncology Faculty of Medicine Mashhad University of Medical Sciences Mashhad Iran; ^4^ Vector‐borne diseases Research Center North Khorasan University of Medical Sciences Bojnurd Iran

**Keywords:** amphotericin B, diagnosis, Iran, leishmaniasis, visceral

## Abstract

Leishmaniasis is a rare complication in adult cases even in endemic areas. Here, the first report of visceral leishmaniasis in a young woman in northeast of Iran has been described.

## Graphical abstract

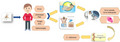

## 1 INTRODUCTION

Leishmaniasis is a main public infectious problem, among visceral leishmaniasis (VL) is the most severe form of the infection which can be lethal in all of the cases if left untreated. Herein, we report a 36‐year‐old woman with enlarged spleen accompanied by prolonged fever and pancytopenia.

Leishmaniasis, a vector‐borne parasitic disease, is caused by intracellular protozoa of more than 20 *Leishmania* species. Infected sandflies vectors transmit the leishmania parasites to humans or the other mammals through their bites.^1^, ^2^ The World Health Organization (WHO) has listed leishmaniasis as one of the most ignored ailments and estimated 350 million people are at the risk of this disease.^3^ Human leishmaniasis is divided into three clinical forms as follows: cutaneous (involve skin), mucocutaneous (involve mucous membranes), and visceral (involving the skin, and visceral organs).^1^, ^4^ Visceral leishmaniasis (VL) is the most severe form of the disease which can be fatal in almost 100% of the cases if left untreated.^1^, ^2^ VL is caused by diverse *Leishmania* species including donovani, Chagas, and infantum related to the certain geographical regions.^4^ Moreover, *Leishmania infantum* is the main cause of VL in the Central Asia, Mediterranean, and Iran. Wide spectrum of symptoms has been reported for this complication ranged from asymptomatic forms to acute and lethal ones.^5^ Usually, VL is diagnosed with atypical fevers, weight loss, anemia, swollen hands and legs, spleen pain, hepatomegaly, and splenomegaly.^6^ So, the early diagnosis of this complication has great importance because of time‐consuming treatment procedures.^4^ VL not only is endemic in rural areas of Iran but also the northwestern region of Iran is reported as a major endemic area of VL.^4^ During 1998‐2012, 2632 VL patients were documented in Iran with the peak incidence in 2000. However, the incidence rate of VL has fallen in recent years.^7^

This is the first report of VL in an adult case in the northeastern region of Iran, who was detected in Imam Hassan Hospital of North Khorasan Province, Bojnurd, Iran, in 2019.

## 2 CASE DESCRIPTION

**Figure 1:**
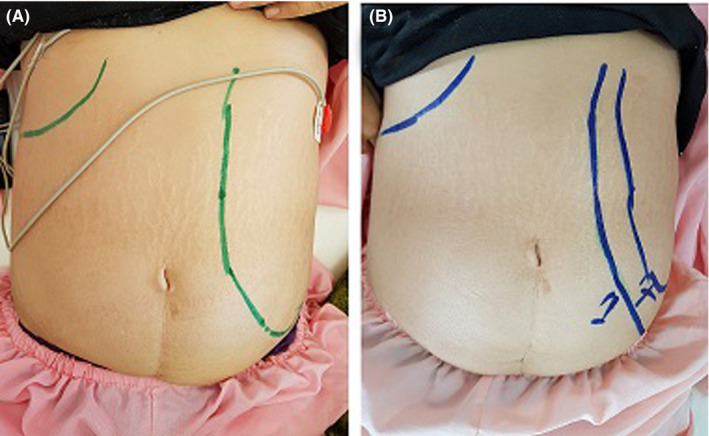
Spleen enlargement in the physical examination of the case. Upon admission (A), one week after treatment (B)

**Figure 2:**
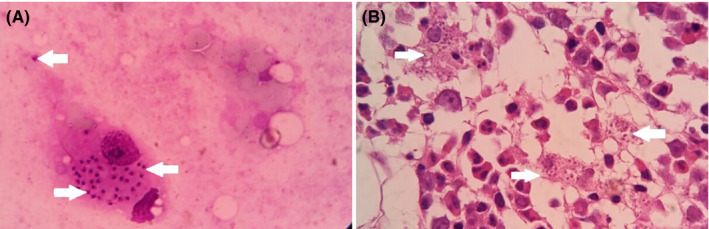
Abundant intracellular *Leishmania* parasite bodies (white arrows), which are small oval or round shaped in bone marrow biopsy (A) and bone marrow aspiration (B)

**Figure 3:**
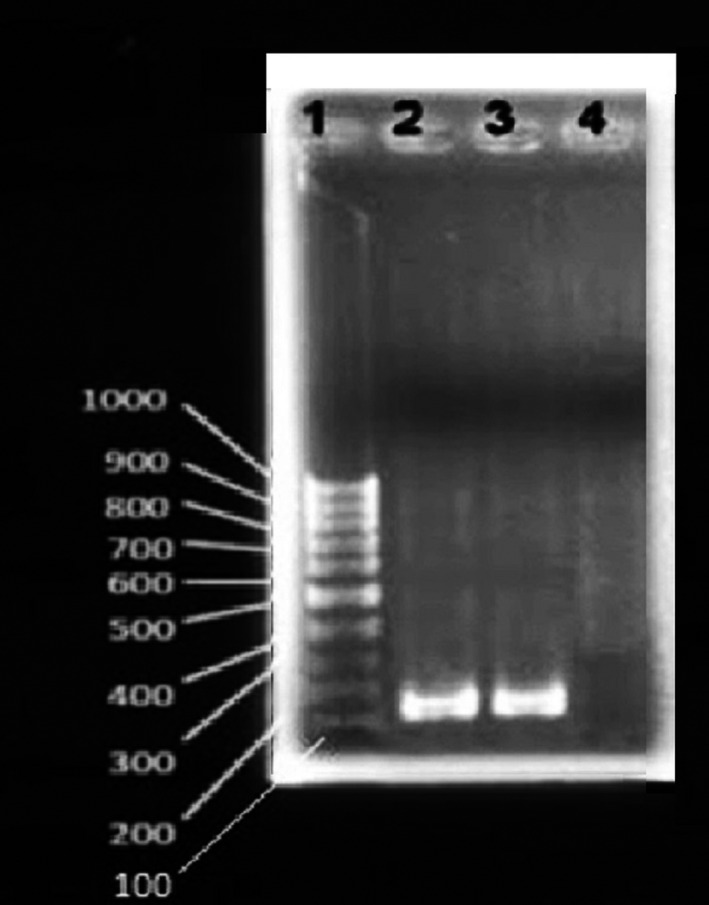
Polymerase chain reaction (PCR) results of *Leishmania infantum* disseminated visceral leishmaniasis (VL) in the patient. Lane 1 represents 10‐bp size marker; lanes 2 indicates *L infantum* in the case; and lane 3 and 4, respectively, demonstrate the positive and negative controls. The primers designed for *L infantum* were as follows: F: 5′‐TCCCAAACTTTTCTGGTCCT‐3′ and R: 5′‐TTACACCAACCCCCAGTTTC‐3′

In March 2019, a 36‐year‐old woman presented to the emergency department of Imam Hassan Hospital with 2 months of prolonged fever, cough, abdominal pain and swelling, fatigue, and an unintentional sever weight loss (about 30‐40 kg). The physical examination was notable for an enlarged spleen in a way that she could not sit easily and was always lying down (Figure 1). The patient had no history of immunodeficiency and did not report any previous illness. The reported case lived in the province (North Khorasan), and there was no immigration in the patient's history. The results of hematological examination showed severe anemia with obvious changes in red blood cell morphology including poikilocytosis, anisocytosis, and hypochromia and severe leukopenia (Table 1). Accordingly, report of abdomen ultrasonography demonstrated splenomegaly extended to the pelvic. Also, the liver was slightly larger than the normal size. Due to the fever and enlarged spleen, bone marrow aspiration and biopsy were performed. The results of bone marrow pathology revealed the typical appearance of *leishmania* amastigotes, each with a nucleus and a kinetoplast within histiocytes (Figure 2). Moreover, the results of PCR confirmed *Leishmania infantum* resulting in the visceral form of leishmaniasis (Figure 3). Upon diagnosis, amphotericin B was injected at 1 mg/kg for about one month. Then, afterward, the patient's symptoms gradually subsided and her general condition improved.

**Table 1:** The results of laboratory blood test of the case upon admission and 1 mo after follow‐up

Test	Before treatment	After treatment	Normal Ranges	Unit
WBC	1.2	14.9	4‐11	10^3^/μL
RBC	2.35	4.50	3.8‐5.1	10^6^/μL
Hemoglobin	5.5	13.4	12‐16	g/dL
Hematocrit	17.4	40.9	35‐47	%
MCH	21.3	29.8	27‐35	U/L
MCHC	28.7	32.8	31‐37	U/L
neutrophil	39	79.5	35‐38	%
Lymphocytes	47	14.4	18‐44	%
Monocytes	19	6.1	4.7‐12.5	%
Platelet count	58	228	150‐440 10^3^	mm
Giant platelets	Were seen	Negative	Negative	mm
Erythrocyte sedimentation rate	75	15	Female <50 y; <20	mm/hour
C‐reactive protein	Positive (+3)	Negative	Negative	Qualitative
Poikilocytosis	Positive (+)	Negative	Negative	Qualitative
Hypochromia	+2	Negative	Negative	Qualitative
Anisocytosis	+2	Negative	Negative	Qualitative

At the end of treatment, the blood cell counts increased; the other serological parameters returned to the normal ranges; and her weight increased (Table 1). Nevertheless, amphotericin B therapy in our case caused life‐threatening ventricular arrhythmias at the end of the treatment period (the last two days of treatment). Due to the completion of the treatment period, amphotericin was discontinued and the last dose of the drug was ignored. Besides, echocardiographic follow‐up showed that left ventricular function returned to the normal state after one week of treatment cessation.

## 3 DISCUSSION

Visceral leishmaniasis (VL), known as “black disease” or “kala‐azar,” is endemic in 98 countries.^8^ The most cases of visceral form reporting from India, Bangladesh, Sudan, Nepal, and Brazil.^9^ Besides, dogs are the main reservoirs, with an important role in transmission of infection to human. With affecting approximately 500,000 cases annually, the disease has a great impact on human life worldwide. The incidence of 100‐300 cases annually makes Iran an endemic region for VL.^10^ In many endemic areas of VL in Iran and other parts of the world, the majority of cases remain asymptomatic.^11^ Visceral leishmaniasis is associated with hematological disorders such as leukopenia, thrombocytopenia, anemia, and pancytopenia. The reason of anemia in VL is related to not only spleen enlargement which sequestrates and destroys red blood cells (RBC) but also immune mechanisms and alterations in permeability of RBCs᾿ membrane.^12^ The principal agent of human and canine VL is *Leishmania infantum*, and *L tropica* is the second etiological ones specially in immunosuppressed individuals.^13^

The zoonotic form of VL caused by *L infantum* (the main agent of VL) is endemic in northwestern and southern area of Iran with the highest incidence rate in Ardabil and East Azerbaijan provinces, northwestern Iran. It has been reported that near 99% of VL cases were seen in children ≤ 12 years old in the VL endemic regions. Furthermore, 95% of the cases were diagnosed in rural areas and 63.1% were found during cold months.^13^ Based, visceral leishmaniasis is more common in children and is rarely seen in adults even in endemic areas. So, it is important to report cases in middle‐aged adults even in endemic areas. In our case (36‐year‐old female), considering the geographical region where patient lived there (northeast of Iran) and the endemicity of kala‐azar in this region, the physician suspected to visceral leishmaniasis and bone marrow puncture performed. The disease was diagnosed based on observing the amastigote shape of parasite in bone marrow aspiration and biopsy (Figure 2) and confirmed with PCR (Figure 3). The results of blood test before treatment showed sever pancytopenia (Table 1). Furthermore, anemia in our case accompanied by blood cell destruction in spleen was associated with splenomegaly. At the end of treatment, VL infection factors such as C‐reactive protein and blood cell counts and indexes backed to the normal ranges (Table 1).

The main complication of VL is coinfection with HIV/AIDS which has become a major health concern all around the world.^8^ The reported case was married and had no history of HIV risk factors. Also, HIV is not very prevalent in the area. So, the dramatic response to treatment and recovery of the patient had not been studied in this regard in our case.

Although near 25 compounds are available for leishmaniasis treatment, a few of them are effective in human cases among pentavalent antimonial agents are used for about 80 years. These compounds including stibogluconate (Pentostam) and meglumine antimoniate (Glucantime) are effective antileishmanial compounds, but their efficacy has reduced because of drug resistance. Besides, these compounds are toxic and associate with adverse side effects.^10^, ^14^ In a randomized clinical trial carried out by Alborzi et al, in south of Iran, Glucantime was used for treatment of VL, and the results showed its efficacy.^8^ The other drugs in VL treatment are amphotericin B, and pentamidine with both acceptable efficacy and toxic side effects. Recently, antifungal azoles and medicinal plant derivatives have been studied in order to find more effective and less toxic agents for treatment of leishmaniasis and promising results have been achieved.^15^, ^16^ Amphotericin B is an antifungal with antileishmanial efficacy. The mechanism of action is trough binding to parasite ergosterols which lead to membranous damage and parasite death. New liposomal formulation of amphotericin B facilitates the drug uptake by macrophages which contains parasite in parasitophorous vacuoles. This formulation is less toxic and more safe with better uptake property and is the only approved formulation by the US food and drug administration (FDA) for the treatment of VL.^17^ Since our case was treated with simple form of amphotericin B for about one month due to the inaccessibility of liposomal form, she encountered with life‐threatening conditions. Fortunately, after only one week all side effects disappeared and she recovered.

## 4 CONCLUSION

With considering VL endemic regions, each patient with anemia and splenomegaly accompanied by prolonged fever may have been infected with *leishmania* parasite. So, a comprehensive examination must be performed for the accurate diagnosis and the best treatment administration should be prescribed based on the patient's tolerance and available medicines. It should be mentioned that amphotericin B injection in VL cases is a challenging process and should be ordered with more cautions.

## CONFLICT OF INTEREST

None declared.

## AUTHOR CONTRIBUTIONS

MRT, MS‐S, AA, MM, MSM, and ZZ: participated in clinical and pathological researches; SM and AS: studied related articles and drafted the manuscript; all authors read and approved the final manuscript.

## ETHICAL APPROVAL

Applicable.

## INFORMED CONSENT

Informed consent was obtained from the patient included in the study.

## References

[1] Alemayehu B , Alemayehu M . Leishmaniasis: A Review on Parasite, Vector and Reservoir Host. Heal Sci J. 2017;11:519.

[2] Azizi MH , Bahadori M , Dabiri S , Shamsi Meymandi S , Azizi F . A History of Leishmaniasis in Iran from 19th Century Onward. Arch Iran Med. 2016;19:153‐162. 26838089

[3] Alvar J , Vélez ID , Bern C , et al. Leishmaniasis Worldwide and Global Estimates of Its Incidence. PLoS One. 2012;7:e35671. 22693548 10.1371/journal.pone.0035671 PMC3365071

[4] Abdinia B , Oliaei‐motlagh M , Teimouri‐dereshki A . Pediatric visceral leishmaniasis in northwest of Iran. Medicine (Baltimore). 2016;95:e5261. 27858891 10.1097/MD.0000000000005261 PMC5591139

[5] Alborzi A , Rasouli M , Shamsizadeh A . Leishmania tropica‐isolated Hyg, patient with visceral leishmaniasis in southern Iran. Am J Trop Med. 2006;74:306‐307. 16474088

[6] Gupta V , Tripathi S , Tilak V , Bhatia BD . A study of clinico‐haematological profiles of pancytopenia in children. Trop Doct. 2008;38:241‐243. 18820199 10.1258/td.2008.070422

[7] Zanjirani Farahani L , Saghafipour A , Mohebali M , Akhoundi B , Raufi H . Visceral leishmaniasis ( Kala‐azar ) in Qom Province, Iran : Report of two cases [version 3; peer review : 2 approved ]. F1000 Res. 2019;7:1‐12. 10.12688/f1000research.15805.1 PMC6593324 31281631

[8] Karimi A , Alborzi A , Amanati A . Visceral leishmaniasis: an update and literature review. Arch Pediatr Infect Dis. 2016;4:e31612.

[9] Thornton SJ , Wasan KM , Piecuch A , Lynd LL , Wasan EK . Barriers to treatment for visceral leishmaniasis in hyperendemic areas: India, Bangladesh, Nepal, Brazil and Sudan. Drug Dev Ind Pharm. 2010;36(11):1312‐1319. 20545513 10.3109/03639041003796648

[10] Shokri A , Fakharb M , Teshnizic SH . Acta Tropica Canine visceral leishmaniasis in Iran: A systematic review and. Acta Trop. 2017;165:76‐89. 27570207 10.1016/j.actatropica.2016.08.020

[11] Layegh Gigloo A , Sarkari B , Rezaei Z , Hatam GR , Davami MH . Asymptomatic Leishmania Infected Children: A Seroprevalence and Molecular Survey in a Rural Area of Fars Province, Southern Iran. J Trop Med. 2018;1‐6. 10.1155/2018/8167247 PMC5976912 29861748

[12] Sarkari B , Naraki T , Ghatee MA , Abdolahi Khabisi S , Davam MH . Visceral Leishmaniasis in Southwestern Iran: A Retrospective Clinico‐Hematological Analysis of 380 Consecutive Hospitalized Cases (1999–2014). PLoS One. 2016;11:e0150406. 26942443 10.1371/journal.pone.0150406 PMC4778872

[13] Mohebali M . Visceral leishmaniasis in Iran: Review of the Epidemiological and Clinical Features. Iran J Parasitol. 2013;8:348‐358. 24454426 PMC3887234

[14] Shokri A , Akhtari J , Keighobadi M , et al. Promising antileishmanial effectiveness of doxorubicin and Doxil against Leishmania major: An in vitro assay. Asian Pac J Trop Med. 2017;10:544‐548. 28756917 10.1016/j.apjtm.2016.09.014

[15] Barati M , Sharifi I , Sharififar F , et al. Extracts by Colorimetric Assay. Anti‐Infective Agents. 2014;12:159‐164.

[16] Shokri A , Emami S , Fakhar M , Teshnizi SH , Keighobadi M . In vitro antileishmanial activity of novel azoles (3‐imidazolylflavanones) against promastigote and amastigote stages of Leishmania major. Acta Trop. 2017;167:73‐78. 28017860 10.1016/j.actatropica.2016.12.027

[17] Chappuis F , Sundar S , Hailu A , et al. Visceral leishmaniasis: what are the needs for diagnosis, treatment and control? Nat Rev Microbiol. 2007;5:873‐882. 17938629 10.1038/nrmicro1748

